# Three Genes Predict Prognosis in Microenvironment of Ovarian Cancer

**DOI:** 10.3389/fgene.2020.00990

**Published:** 2020-09-02

**Authors:** Ya Guo, Ya Li Wang, Wang Hui Su, Peng Tao Yang, Jing Chen, Heng Luo

**Affiliations:** Department of Radiation Oncology, The Second Affiliated Hospital, Xi’anjiao Tong University, Xi’an, China

**Keywords:** ovarian cancer, tumor microenvironment, The Cancer Genome Atlas, immune score, stromal score, overall survival

## Abstract

Ovarian cancer (OC) is the deadliest gynecological cancer in women. Immune cell infiltration has a critical role in regulating carcinogenesis and prognosis in OC. To identify prognostic genes relevant to the tumor microenvironment in OC, we investigated the association between OC and gene expression profiles. Results obtained with the ESTIMATE R tool showed that immune score and stromal score were correlated with lymphatic invasion, and high immune score predicted a favorable prognosis. A total of 342 common differentially expressed genes were identified according to the two scores; these genes were mainly involved in immune response, extracellular region, and serine-type endopeptidase activity. Three immune-related prognostic genes were selected by univariate and multivariate Cox regression analysis. We further established a prognostic model and validated the prognostic value of three hub genes in different databases; our results showed that this model could accurately predict survival and evaluate prognosis independent of clinical characteristics. Three hub genes have prognostic value in OC. TIMER analysis revealed that the three genes were correlated with different immune cells. Low levels of macrophage infiltration and high levels of CD4+ T cell infiltration were associated with favorable survival outcomes. Arm-level gain of GYPC was correlated with neutrophils and dendritic cells. These findings indicate that CXCR4, GYPC, and MMP12 modulate prognosis via effects on the infiltration of immune cells. Thus, these genes represent potential targets for immune therapy in OC.

## Introduction

Ovarian cancer (OC) is the eighth most prevalent cancer and the deadliest gynecological malignancy. Early diagnosis and treatment are associated with favorable prognosis. However, in more than two-thirds of patients, this cancer is diagnosed at a late stage, resulting in a poor prognosis (Guo et al., 2019). Although surgery, chemotherapy, molecular targeted drugs, and immunotherapy have led to rapid advances, the cure rate of patients with advanced OC remains poor, with a 5-year survival rate of approximately 30% (Jiang et al., 2020; Shimizu et al., 2020). Therefore, the effective biomarkers are needed to improve the prognosis of patients with OC.

Accumulating evidence indicates that the tumor microenvironment (TME) has significant clinical value in predicting prognosis (Pu et al., 2019; Hong et al., 2020; Ling et al., 2020; Qu et al., 2020). Previous studies indicated that T cells were positively correlated with good clinical outcomes in OC (Fridman et al., 2012). Infiltration of B cells was reported to be linked to excellent prognosis in epithelial OCs (Giraldo et al., 2014). In glioblastoma, the TME is strongly linked to gene expression and prognosis (Cooper et al., 2012). In addition, cancer-associated fibroblasts (CAFs) enhance OC metastasis and induce upregulation of lipoma-preferred partner, which contributes to chemoresistance in OC (Ding et al., 2020).

Owing to advances in sequencing technologies, large amounts of bioinformatic data are available from public databases, including The Cancer Genome Atlas (TCGA) and the Gene Expression Omnibus (GEO) database, enabling exploration of the association between TME and prognosis in tumors (Zhang et al., 2019). The recently developed ESTIMATE algorithm can predict infiltration of non-tumor cells by evaluating specific gene expression in immune and stromal cells (Ding et al., 2020). The ESTIMATE algorithm has been applied to various cancers, including prostate cancer, breast cancer, colon cancer, cutaneous melanoma, glioblastoma, and clear cell renal cell carcinoma (Piperi et al., 2019; Bangbei et al., 2020; Yang H. et al., 2020; Yang S. et al., 2020; Zhang Z. et al., 2020). However, there has been limited research on the TME in OC, and the mechanism by which the TME influences prognosis in OC remains unclear. This study aimed to identify prognosis-related genes and clarify the underlying mechanism linking the TME and prognosis in OC. Data from 593 OC patients were obtained from TCGA; we calculated immune or stromal scores for these patients using the ESTIMATE algorithm and used these scores to divide patients into low and high immune/stromal score groups. Differentially expressed genes (DEGs) were identified between the two groups. Functional annotations of these DEGs were analyzed using Database for Annotation, Visualization and Integrated Discovery (DAVID)^1^ (Huang et al., 2009). Moreover, prognostic DEGs were identified by comprehensive analysis. Finally, we analyzed immune infiltration, the association between somatic copy number alterations (SCNAs) and immune infiltrates, and the prognosis of various immune cells using the Tumor Immune Estimation Resource (TIMER)^2^ (Hu et al., 2020).

## Materials and Methods

### Database

Gene expression profiles and clinical information for OC were downloaded from TCGA using the UCSC Xena Browser^3^ (Pu et al., 2019; Du et al., 2020; Zhang Z. et al., 2020), and TCGA ovarian carcinoma gene expression data were obtained using the AffyU133a array. Scores were computed with the ESTIMATE algorithm^4^.

### Identification of DEGs Based on Different Immune Scores and Stromal Scores

Differential expression analysis of the data was performed using the limma package (Yan et al., 2019; Xie et al., 2020). DEGs were selected using fold change > 1.5 and adj. *P* < 0.05. The ClustVis web tool^5^ was used to generate heat maps and for clustering (Metsalu and Vilo, 2015). The jvenn software^6^ was used to display shared upregulated and downregulated genes between the two score groups (Bardou et al., 2014; Liu et al., 2020).

## Functional Enrichment Analysis of 342 Intersecting Genes

Gene ontology (GO) analysis of the 342 common DEGs was performed using DAVID (see “footnote 1”) (Huang et al., 2009). GO functional enrichment was determined using the criterion of false discovery rate (FDR) less than 0.05 (Zhang Z. et al., 2020).

### Selection of Prognostic DEGs

Univariate Cox regression was used for preliminary identification of prognostic DEGs (Wu et al., 2020), followed by multivariate analysis to estimate the prognostic value of the identified DEGs (Zhang R. et al., 2020). To construct a risk assessment system, the risk score for each patient was calculated according to regression coefficients and mRNA expression levels (Cai et al., 2019). Based on the median risk score, patients were allocated to high- and low-risk groups (Qian et al., 2020). Kaplan–Meier (KM) survival curves were constructed to evaluate the prognostic value of the DEGs. To estimate the prognostic value of the risk score model, we plotted 3- and 5-year receiver operating characteristic (ROC) curves and calculated the area under the curve (AUC) using the survival and time R packages. Finally, univariate and multivariate independent factor prognostic analyses were used to assess whether the signature could predict patient prognosis independent of clinical characteristics such as age, grade, stage, lymphatic invasion, and subdivision (Hu et al., 2020).

### Validation of the Prognostic Value of Three Hub Genes

PROGgenesV2^7^ is a web based tool that allows researchers to study the association between genes and overall survival (OS) in multiple cancers based on TCGA and GEO data (Goswami and Nakshatri, 2014). PrognoScan provides a powerful platform for evaluating the relation between gene expression and patient prognosis such as OS and disease-free survival (DFS) across a large collection of publicly available cancer microarray datasets. The database is publicly accessible at http://www.prognoscan.org/ (Mizuno et al., 2009). Online consensus Survival for Ovarian Cancer (OSov) encompasses 22 expression datasets and provides six types of survival terms for 3212 patients of OC, which can be available at http://bioinfo.henu.edu.cn/OV/OVList.jsp (Zheng et al., 2020). In this study, PROGgenesV2, PrognoScan, and OSov database was used to assess the prognostic value of hub genes. *P* ≤ 0.05 was considered as significant.

### Association Between Prognostic Signature and Immune Cell Infiltration

Tumor Immune Estimation Resource contains seven modules: gene, survival, mutation, SCNA, diff exp, correlation, and estimation (Hu et al., 2020). In this study, we used the gene, survival, and SCNA modules to evaluate the association between prognostic biomarkers and immune cell infiltration.

### Statistical Analysis

Univariate analysis and multivariate Cox regression analyses were performed using the R survival package. Risk plots and risk survival curves were analyzed and visualized using the survival and pheatmap packages. All these analyses used R version 3.6.3^8^ (Luan et al., 2019).

## Results

### Immune Scores and Stromal Scores Are Related to Clinical Factors in OC

**FIGURE 1:**
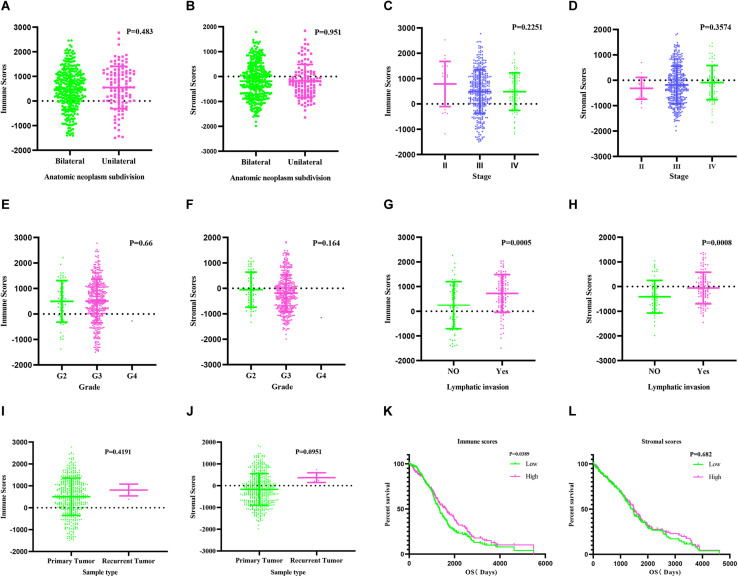
Immune and stromal scores are correlated with ovarian cancer clinical characters and their overall survival. **(A,B)** Distribution of immune and stromal scores of ovarian cancer anatomic neoplasm subdivision. **(C,D)** Distribution of immune and stromal scores of ovarian cancer stage. **(E,F)** Distribution of immune and stromal scores of ovarian cancer grade. **(G,H)** Distribution of immune and stromal scores of ovarian cancer lymphatic invasion. **(I,J)** Distribution of immune and stromal scores of ovarian cancer sample type. **(K)** Kaplan–Meier (KM) overall survival (OS) curve for high and low immune score group. **(L)** KM survival curve showing the OS time of high and low stomal score group.

We estimated immune score and stromal score based on gene expression data using the ESTIMATE algorithm (Xie et al., 2019; Mahal et al., 2020; Pan et al., 2020). Both scores were correlated with lymphatic invasion (*P* = 0.0005 and *P* = 0.0008, respectively). There were no associations between the scores and other clinical factors, including anatomic neoplasm subdivision, grade, sample type, and stage (Figures 1A–J). KM survival analysis demonstrated that patients with low immune scores had poor prognosis (*P* = 0.0389) (Figure 1K). The stromal scores showed no association with OS (Figure 1L).

### Identification of DEGs

**FIGURE 2:**
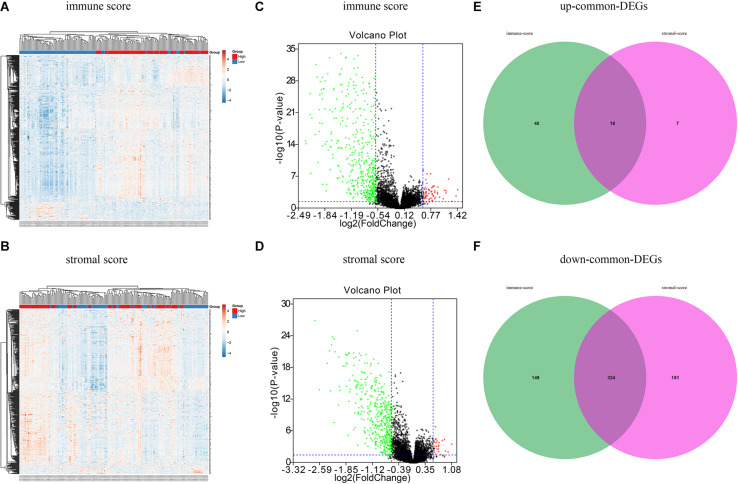
Different expression genes based on immune scores and stromal scores in ovarian cancer. **(A)** Heat maps demonstrated the different expression genes (DEGs) between high immune scores low immune scores. *P* < 0.05, fold change > 1.5. **(B)** Heat map of DEGs between high stromal scores and low stromal scores. *P* < 0.05, fold change > 1.5. Red module represents high-expression genes; low-expression genes are shown in blue. **(C)** Volcano plot illustrated the DEGs based on immune scores. **(D)** Volcano plot displayed the DEGs based on stromal scores. Red dots represent upregulated genes, green dots represent downregulated genes. **(E)** Venn diagrams displayed the commonly upregulated genes in immune and stromal groups. **(F)** Venn diagrams showed the commonly downregulated genes.

A total of 593 OC patients were classified into high and low immune/stromal score groups. To determine the association between gene expression and immune and stromal scores, we compared gene expression profiles between groups. As shown in Figure 2A, 531 DEGs were detected in the high immune score group, including 58 upregulated genes and 473 downregulated genes (fold change > 1.5, *P* < 0.05), and 500 DEGs were obtained in the high stromal score group, including 25 upregulated and 475 downregulated genes (fold change > 1.5, *P* < 0.05) (Figure 2B). Volcano plots (Figures 2C,D) were used to display the distribution of the DEGs in the two score groups. According to the Venn analysis, there were 342 common genes, including 324 downregulated DEGs and 18 upregulated DEGs (Figures 2E,F).

### Functional Annotation of 342 Intersecting Genes

**FIGURE 3:**
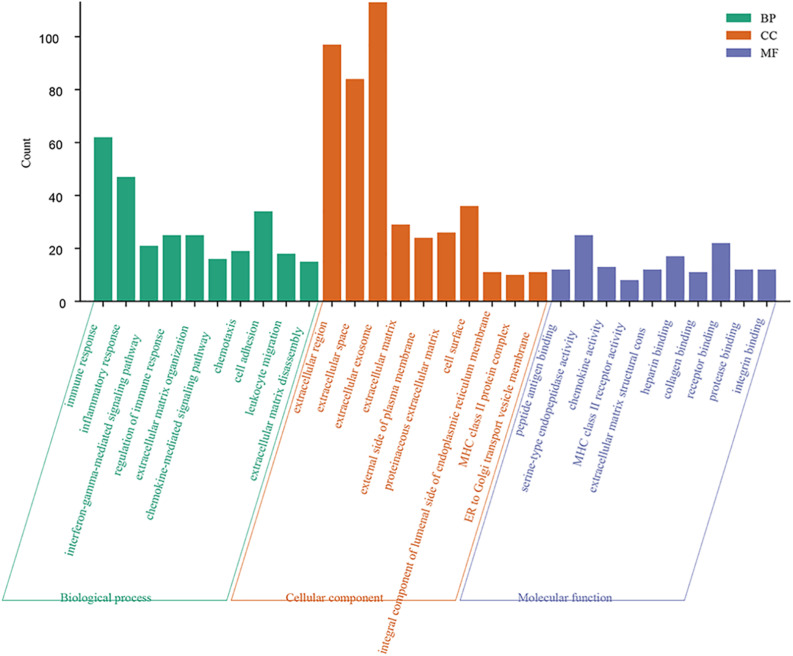
Function enrichment analysis of 342 intersection genes. Top 10 enriched GO terms of 342 common DEGs. Significantly GO terms were identified using DAVID functional annotation tool (https://david.ncifcrf.gov/). FDR smaller than 0.05 was used as the criteria for significantly enriched. Enriched GO terms included three categories: biological process, cellular component, and molecular function.

Forty-four GO terms in the biological process category were significantly enriched. The top five biological process GO terms were immune response, inflammatory response, interferon-gamma-mediated signaling pathway, regulation of immune response, and extracellular matrix organization. A total of 19 cellular component GO terms were enriched; these were mainly related to the extracellular region. Eleven GO terms in the molecular function category were significantly overrepresented (FDR < 0.05) (Figure 3).

### Identification of Prognostic Signature and Evaluation of Prognostic Model

**FIGURE 4:**
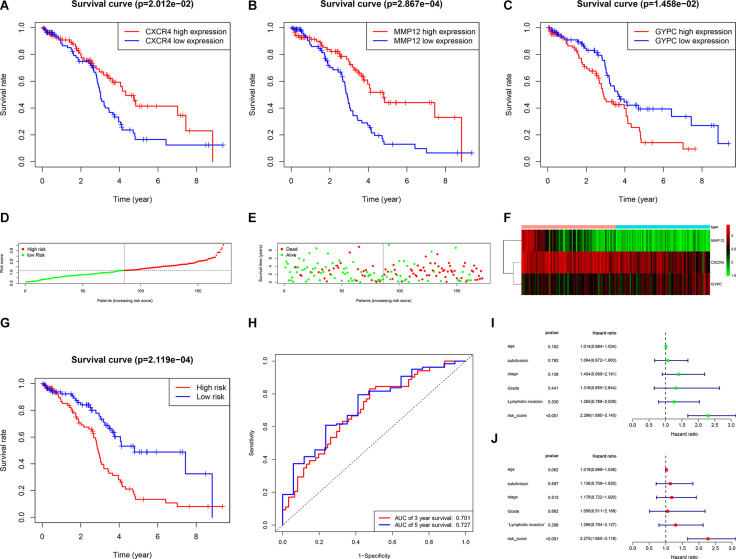
Establishment and evaluation prognostic model. **(A–C)** Survival rates were calculated between high and low gene expression groups. The red line represents the high gene expression group, and the blue line represents the low gene expression group. **(A)** CXCR4, **(B)** MMP12, **(C)** GYPC. **(D–F)** Distribution of risk score ranking, survival status, and three DEGs’ expression heat map. **(G)** Survival curves of patients in the high-risk group and low-risk group. **(H)** 3- and 5-year ROC curves. ROC, receiver operating characteristic; AUC, area under the curve. **(I,J)** Univariate and multiple regression analysis was performed to assess the relationship between age, grade, stage, lymphatic invasion, subdivision, and risk scores.

We first conducted univariate Cox regression analysis to identify prognostic genes among the 342 common DEGs. Our result showed that seven DEGs had prognostic value for OC (Table 1). We next performed multivariate Cox regression analysis on these seven DEGs to identify a prognostic signature in OC and obtained three DEGs that constituted a prognostic signature for OC. Survival curves were plotted according to the expression levels of these three DEGs. CXCR4 and MMP12 were identified as favorable prognostic factors, whereas GYPC has poor predictive value (Figures 4A–C). Moreover, we constructed a risk score model for predicting prognosis, which included risk score ranking, survival status, and a heat map of the three DEGs’ expression levels (Figures 4D–F). We further plotted survival curves for the high- and low-risk groups. As shown in Figure 4G, the survival rate of patients in the high-risk groups was lower than the survival rate of those in the low-risk groups (*P* = 0.00021). Besides, the 3- and 5-year AUC values were 0.701 and 0.727, respectively, indicating that the risk score model can effectively evaluate prognosis (Figure 4H). Univariate and multivariate independent prognostic analyses were used to assess whether the risk score was independent of other clinical characteristics as an evaluation factor. Our results demonstrated that the risk score could accurately predict survival, regardless of the influence of age, grade, stage, lymphatic invasion, and subdivision; thus, it represents an independent prognostic factor for poor survival in OC (Figures 4I,J).

**TABLE 1:** Identification of prognosis related signature by Univariate Cox Regression.

Gene	HR	HR.95L	HR.95H	*P* value
BCL2A1	0.72621	0.53674	0.98256	0.03808
CCL18	0.66186	0.47817	0.91612	0.01284
CXCR4	0.72925	0.59483	0.89404	0.00239
GYPC	1.24800	1.01074	1.54096	0.03947
MMP12	0.65599	0.52586	0.81833	0.00019
MMP9	0.80185	0.69033	0.93139	0.00385
UBD	0.90017	0.81083	0.99934	0.04858

### Three Hub Genes Have Prognostic Value in OC

To further confirm the prognostic value of the identified three hub genes, the association between three hub genes and prognosis was analyzed using different databases, including PROGgenesV2, PrognoScan, and OSov. The results indicated that high GYPC expression might be a candidate for poor prognosis in OC patients, and MMP12 expression was positively correlated with good OS and PFS. Based on TCGA dataset, high expression of CXCR4 predicts a favorable prognosis. These results are consistent with our results. However, in GSE8842, GSE13867, and GSE30161, a positive CXCR4 expression indicated a poor prognosis. In summary, these results suggested that three hub genes may be promising biomarkers that predict the prognosis of all OC patients (Table 2).

**TABLE 2:** Confirmation of prognostic value of three hub genes.

Database	Datasets	Gene Symbol	Endpoint	HR [95% CI-low CI-upper]	*P* value
PROGgeneV2	GSE8842	CXCR4	OS	2.18 [1.12−4.26]	0.0225
PROGgeneV2	TCGA	CXCR4	OS	0.9 [0.82−0.99]	0.0271
OSov	GSE13867	CXCR4	OS	1.3124 [1.0215−1.6862]	0.0335
OSov	GSE30161	CXCR4	OS	2.1406 [1.0143−4.5175]	0.0458
OSov	TCGA	CXCR4	OS	0.7282 [0.5308−0.9989]	0.0492
PROGgeneV2	GSE19829_U95V2	GYPC	OS	5.63 [1.49−21.35]	0.0110
PROGgeneV2	GSE26712	GYPC	OS	1.56 [1.06−2.29]	0.0250
PrognoScan	GSE26712_202947_s_at	GYPC	OS	1.46 [1.00−2.13]	0.0499
PROGgeneV2	GSE32062	MMP12	OS	0.92 [0.86−0.99]	0.0171
PROGgeneV2	TCGA	MMP12	OS	0.89 [0.82−0.96]	0.0024
PrognoScan	GSE17260_A_23_P150316	MMP12	PFS	0.80 [0.67−0.94]	0.0066
PrognoScan	GSE17260_A_23_P340698	MMP12	PFS	0.87 [0.78−0.96]	0.0079
OSov	GSE3149	MMP12	OS	0.5339 [0.2937−0.9707]	0.0396

*PROGgeneV2, OSov, PrognoScan databases were used to validate the correlation between the three hub genes and prognosis in OC. HR = Hazara Ratio, Progression Free Survival = PFS, Overall Survival = OS.*

### Evaluation of the Associations Among the Three Prognostic Genes, Immune Cells, and Survival

**FIGURE 5:**
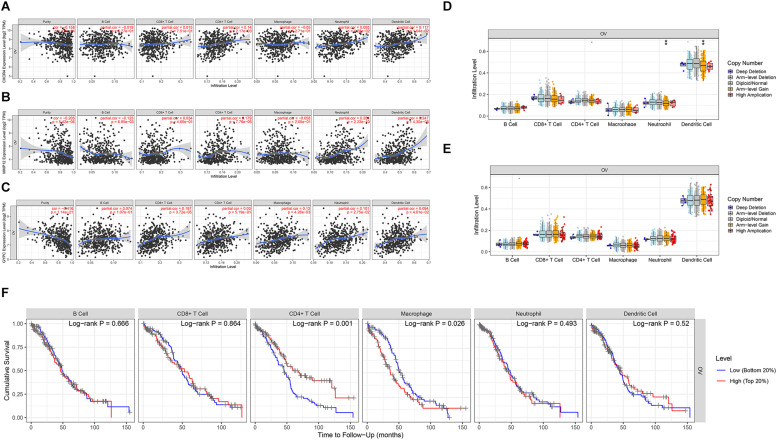
Evaluation of the association between three prognostic signatures, immune cells, and survival. Three prognostic signatures associated with immune cell infiltration. **(A)** CXCR4, **(B)** MMP12, **(C)** GYPC. **(D,E)** Association of three prognostic signature mutants with immune cell infiltration. **(D)** The arm level gain of GYPC was correlated with neutrophils and dendritic cells. **(E)** The SCNA level of MMP12 was not significant with immune infiltration. Association between CXCR4 mutants with immune cell infiltration was not detected by the SCNA module of timer. **(F)** Survival analysis of differentially immune cells. Low infiltration level of macrophage cells and high infiltration level of CD4+ T cells correlated with favorable survival outcomes. Statistical significance was defined by a *P* value: *** means 0 ≤ *P* value < 0.001, ** represents 0.001 ≤ *P* value < 0.01, * represents 0.01 ≤ *P* value < 0.05, means 0.05 ≤ *P* value < 0.1.

We assessed the association between the three prognostic biomarkers and immune cell infiltration using TIMER. The results showed that the expression of CXCR4 was related to CD4+ T cells, neutrophils, and dendritic cells. GYPC was related to four immune cell types: CD8+ T cells, macrophages, neutrophils, and dendritic cells. MMP12 was primarily related to B cells, CD4+ T cells, neutrophils, and dendritic cells (Figures 5A–C). We further used the SCNA module for comparison of tumor infiltration levels among tumors with different SCNAs for the three prognostic biomarkers. Six immune cell types displayed significant downregulation in OC associated with SCNA of GYPC; arm-level gain of GYPC was correlated with neutrophils and dendritic cell. The SCNA level of MMP12 was not significantly associated with immune infiltration (Figures 5D,E). In addition, we used the survival module of TIMER to examine the relationship between clinical characteristics and immune cell infiltration. Our results showed that infiltration levels of CD4+ T cells and macrophages could predict patient survival in OC. High infiltration levels of macrophages and low infiltration levels of CD4+ T cells were associated with poor survival (Figure 5F).

## Discussion

Ovarian cancer is among the most prevalent cancers in women (Cheng et al., 2020). The TME has a major impact on tumor progression, recurrence, and metastasis, resulting in poor prognosis (Chen et al., 2019; Huang et al., 2019; Zhang F.-P. et al., 2020). Previous studies have shown that the TME influences initiation and progression of OC and affects anti-tumor treatment (Jiang et al., 2020). However, the effects of genes associated with the TME on the prognosis of OC patients are still poorly understood. Therefore, identification of prognostic genes related to the TME is critical to enhance the survival of patients with OC.

Lymphatic invasion is associated with poor prognosis in OC patients (Matsuo et al., 2012). In the present study, a comprehensive analysis of DEGs in the TME was performed, and associations among DEGs, prognosis, and immune cells were investigated. High immune and stromal scores were associated with lymphatic invasion (*P* = 0.0005, *P* = 0.0008), and patients with high immune scores had longer OS (*P* = 0.0389). Our data suggest that the clinical outcomes of OC patients could be influenced by their TME type (Figures 1A–K).

We next compared gene expression profiles between the different immune/stromal score groups. A total of 342 common genes were identified, including 18 upregulated genes and 324 downregulated genes. GO term analysis resulted in 342 common genes, mainly involved in the TME. The top five biological process GO terms were immune response, inflammatory response, interferon-gamma-mediated signaling pathway, regulation of immune response, and extracellular matrix organization (Figure 3). Our results suggest that DEGs associated with the TME might contribute to the development of OC by affecting the above mentioned biological process.

We further performed univariate and multivariate Cox regression analysis to identify prognostic TME-related genes. Three downregulated DEGs (CXCR4, MMP12, and GYPC) were identified as a prognostic signature for OC, of which CXCR4 and MMP12 were recognized as favorable prognostic factors. GYPC was regarded as a poor prognostic factor (Figures 4A–C). Three databases were carried out to assess the prognostic value of three hub genes. The results indicated high expression of MMP12 and low GYPC expression predicts favorable prognosis in TCGA and GEO datasets. In TCGA dataset, the expression of CXCR4 is positively related to favorable prognosis. These results are in agreement with our results. However, in the GEO dataset, a positive CXCR4 expression was correlated with a poor prognosis. This different result may be related to different statistical methods and sample composition. CXCR4 is a G-protein-coupled chemokine receptor that is upregulated in many cancers, including thyroid, breast, pancreatic, prostate, and kidney cancers, renal cell carcinoma, and OC (Du et al., 2015; Gong et al., 2020). Previous studies showed that upregulation of CXCR4 was strongly correlated with poor prognosis in renal cell carcinoma (Du et al., 2015). In OC, inhibiting the expression of CXCR4 can prevent cell proliferation and promote cell apoptosis, possibly via the MAPK signaling pathway (Wang et al., 2018). CXCR4 affects OC progression through enhancing tumor angiogenesis and suppressing immunity/inhibiting immunity (Gil et al., 2014). A previous meta-analysis showed that high CXCR4 expression was linked to poor prognosis in OC (Liu et al., 2014). Our results are inconsistent with this previous study, indicating that further investigation of the underlying mechanism is required. MMP12 is significantly upregulated in gallbladder cancer, in which it has been identified as a novel prognostic biomarker (Zhao et al., 2019). A meta-analysis and systematic review indicated that MMP12 rs2276109 might have an impact on OC risk (Zhu and Sun, 2017). The G allele of the MMP12 82A/G polymorphism might promote development and progression of epithelial ovarian carcinoma (Li et al., 2009). No association between MMP12 and prognosis in OC has been reported, nor has the role of GYPC in OC been elucidated. In this study, we first identified MMP12 as a favorable prognostic biomarker and GYPC as a negative prognostic biomarker. Taken together, these genes could represent potential prognostic biomarkers for OC.

Increasing evidence indicates that B cells, CAFs, CD4+ T cells, CD8+ T cells, neutrophils, dendritic cells, macrophages, and other cell types have critical roles in OC (Jiang et al., 2020). In OC, CXCL14 secreted by CAFs stimulates LINC0009 to promote OC growth and invasion (Curtis et al., 2019; Jiang et al., 2020). Moreover, CAFs may affect autophagy in OC cells through a series of pro-inflammatory cytokines, autophagy-derived substrates, and metabolites, leading to OC progression (Thuwajit et al., 2018; Jiang et al., 2020). In high-grade serous OC, high levels of infiltration of CD8+ T cells are usually associated with favorable outcomes (Goode et al., 2017). CD8+ T cells and CD4+ T cells both have prognostic value in OC (Hamanishi et al., 2011). In OC, tumor-associated macrophages are believed to involve in immune inhibition and have a critical role in tumor cell invasion, angiogenesis, metastasis, and early relapse (Yin et al., 2016; Drakes and Stiff, 2018). A previous study showed that dendritic cells have a crucial role in initiating and modulating immune responses and are associated with patient outcomes. Mature dendritic cells are linked to increased immune infiltration and improve the prognosis of patients with OC (Truxova et al., 2018). OC cells can impair dendritic cells’ activation, antigen presentation, and differentiation (Jiang et al., 2020). Formation of neutrophil extracellular traps is associated with improved OS in advanced-grade OC (Muqaku et al., 2020). A meta-analysis suggested that the neutrophil-to-lymphocyte ratio could predict OS and progression-free survival of OC patients (Chen et al., 2018; Yin et al., 2019). The DEGs identified in our study were associated with B cells, CAFs, CD4+ T cells, CD8+ T cells, neutrophils, macrophages, dendritic cells, endothelial cells, natural killer cells, and others, consistent with previous studies (Figures 5A–C). Low macrophage infiltration and high CD4+ T cell infiltration are associated with favorable survival outcomes in OC (Figure 5F). This study confirmed the role of CD4+ T cells in positive outcomes.

We further explored the association between expression of the three prognostic genes and immune cell infiltration. CXCR4 and MMP12 were positively correlated with infiltration of CD4+ T cells, neutrophils, and dendritic cells. GYPC was related to CD8+ T cells, macrophages, neutrophils, and dendritic cells. SCNA of GYPC was downregulated in various immune cells, and arm-level gain of GYPC was associated with neutrophils and dendritic cells (Figures 5D,E). CXCR4 was related to altered immune cells, which can affect the prognosis of gastric cancer (Li et al., 2020). A previous study showed that CXCR4 was upregulated in naive CD4+ and CD8+ T cells and CD4+ central memory T cells, which have a crucial role in modulating immune dysfunction (Ramonell et al., 2017). Macrophages express matrix metalloproteinases, which can promote vascularization (Drakes and Stiff, 2018). These results suggest that CXCR4 might modulate the prognosis of OC via infiltration of CD4+ T cells. GYPC was identified as a poor prognostic factor, which might be linked to macrophages, neutrophils, and dendritic cells. The prognostic value of MMP12 was correlated with various immune cell types. The mechanisms underlying these associations require further investigation.

## Conclusion

In conclusion, CXCR4, GYPC, and MMP12 appear to affect prognosis via influencing the infiltration of immune cells; therefore, these genes represent potential targets for immune therapy in OC.

## Data Availability Statement

The RNAseq data (level 3) and clinical information of ovarian cancer samples can be found in UCSC Xena (http://xena.ucsc.edu/).

## Author Contributions

YG designed the study and wrote the main text of the manuscript. YW performed the data analysis. WS reviewed the manuscript. PY performed the language editing. JC and HL prepared the figures and the tables. All authors contributed to the article and approved the submitted version.

## Conflict of Interest

The authors declare that the research was conducted in the absence of any commercial or financial relationships that could be construed as a potential conflict of interest.

## Footnotes

1 https://david.ncifcrf.gov/

2 https://cistrome.shinyapps.io/timer/

3 https://xenabrowser.net/datapages/

4 https://bioinformatics.mdanderson.org/estimate/index.html

5 https://biit.cs.ut.ee/clustvis/

6 http://jvenn.toulouse.inra.fr/app/example.html

7 http://genomics.jefferson.edu/proggene/filter.php

8 https://www.r-project.org/

## References

[B1] Bangbei W. Bo L. Yuan H. Cai L. (2020). Identification of genes of prognostic value in the ccRCC microenvironment from TCGA database. *Mol. Genet. Genom. Med.* 8:e1159. 10.1002/mgg3.1159 32012488 PMC7196483

[B2] Bardou P. Mariette J. Escudié F. Djemiel C. Klopp C. (2014). jvenn: an interactive venn diagram viewer. *BMC Bioinform.* 15:293. 10.1186/1471-2105-15-293 25176396 PMC4261873

[B3] Cai W. Li Y. Huang B. Hu C. (2019). Esophageal cancer lymph node metastasis-associated gene signature optimizes overall survival prediction of esophageal cancer. *J. Cell. Biochem.* 120 592–600. 10.1002/jcb.27416 30242875

[B4] Chen B. Chen W. Jin J. Wang X. Cao Y. He Y. (2019). Data mining of prognostic microenvironment-related genes in clear cell renal cell carcinoma: a study with TCGA database. *Dis. Mark.* 2019:8901649. 10.1155/2019/8901649 31781309 PMC6875323

[B5] Chen G. Zhu L. Yang Y. Long Y. Li X. Wang Y. (2018). Prognostic role of neutrophil to lymphocyte ratio in ovarian cancer: a meta-analysis. *Technol. Cancer Res. Treat.* 17:1533033818791500. 10.1177/1533033818791500 30145940 PMC6111397

[B6] Cheng H. Wang Z. Cui L. Wen Y. Chen X. Gong F. (2020). Opportunities and challenges of the human microbiome in ovarian cancer. *Front. Oncol.* 10:163. 10.3389/fonc.2020.00163 32133297 PMC7040031

[B7] Cooper L. A. D. Gutman D. A. Chisolm C. Appin C. Kong J. Rong Y. (2012). The tumor microenvironment strongly impacts master transcriptional regulators and gene expression class of glioblastoma. *Am. J. Pathol.* 180 2108–2119. 10.1016/j.ajpath.2012.01.040 22440258 PMC3354586

[B8] Curtis M. Kenny H. A. Ashcroft B. Mukherjee A. Johnson A. Zhang Y. (2019). Fibroblasts mobilize tumor cell glycogen to promote proliferation and metastasis. *Cell Metab.* 29 141–155.e149. 10.1016/j.cmet.2018.08.007 30174305 PMC6326875

[B9] Ding Q. Dong S. Wang R. Zhang K. Wang H. Zhou X. (2020). A nine-gene signature related to tumor microenvironment predicts overall survival with ovarian cancer. *Aging* 12 4879–4895. 10.18632/aging.102914 32208363 PMC7138578

[B10] Drakes M. L. Stiff P. J. (2018). Regulation of ovarian cancer prognosis by immune cells in the tumor microenvironment. *Cancers* 10:302. 10.3390/cancers10090302 30200478 PMC6162424

[B11] Du W. Liu T. Zhang Y. Zeng Y. Zhu J. Tang H. (2020). MiR-195-5p is a potential factor responsible for CPNE1 differential expression between subtypes of non-small cell lung cancer. *J. Cancer* 11 2610–2620. 10.7150/jca.39884 32201531 PMC7066018

[B12] Du Y. Long Q. Guan B. Mu L. (2015). Prognostic value of high CXCR4 expression in renal cell carcinoma: a system review and meta-analysis. *Dis. Mark.* 2015:568980. 10.1155/2015/568980 26526157 PMC4615221

[B13] Fridman W. H. Pagès F. Sautès-Fridman C. Galon J. (2012). The immune contexture in human tumours: impact on clinical outcome. *Nat. Rev. Cancer* 12 298–306. 10.1038/nrc3245 22419253

[B14] Gil M. Komorowski M. P. Seshadri M. Rokita H. McGray A. J. R. Opyrchal M. (2014). CXCL12/CXCR4 blockade by oncolytic virotherapy inhibits ovarian cancer growth by decreasing immunosuppression and targeting cancer-initiating cells. *J. Immunol.* 193 5327–5337. 10.4049/jimmunol.1400201 25320277 PMC4225176

[B15] Giraldo N. A. Becht E. Remark R. Damotte D. S-Fridman C. S. HFridman W. (2014). The immune contexture of primary and metastatic human tumours. *Curr. Opin. Immunol.* 27 8–15. 10.1016/j.coi.2014.01.001 24487185

[B16] Gong G. Lin T. Yuan Y. (2020). Integrated analysis of gene expression and DNA methylation profiles in ovarian cancer. *J. Ovar. Res.* 13:30 10.1186/s13048-020-00632-639 PMC7082962 32192517

[B17] Goode E. L. Block M. S. Kalli K. R. Vierkant R. A. Chen W. Fogarty Z. C. (2017). Dose-response association of CD8+ Tumor-infiltrating lymphocytes and survival time in high-grade serous ovarian cancer. *JAMA Oncol.* 3:e173290. 10.1001/jamaoncol.2017.3290 29049607 PMC5744673

[B18] Goswami C. P. Nakshatri H. (2014). PROGgeneV2: enhancements on the existing database. *BMC Cancer* 14:970. 10.1186/1471-2407-14-970 25518851 PMC4300843

[B19] Guo J. Yang W.-L. Pak D. Celestino J. Lu K. H. Ning J. (2019). Osteopontin, macrophage migration inhibitory factor and anti-interleukin-8 autoantibodies complement CA125 for detection of early stage ovarian cancer. *Cancers* 11:596. 10.3390/cancers11050596 31035430 PMC6562667

[B20] Hamanishi J. Mandai M. Abiko K. Matsumura N. Baba T. Yoshioka Y. (2011). The comprehensive assessment of local immune status of ovarian cancer by the clustering of multiple immune factors. *Clin. Immunol.* 141 338–347. 10.1016/j.clim.2011.08.013 21955569

[B21] Hong W. Yuan H. Gu Y. Liu M. Ji Y. Huang Z. (2020). Immune-related prognosis biomarkers associated with osteosarcoma microenvironment. *Cancer Cell Intern.* 20:83 10.1186/s12935-020-1165-1167 PMC7075043 32190007

[B22] Hu B. Yang X.-B. Sang X.-T. (2020). Development of an immune-related prognostic index associated with hepatocellular carcinoma. *Aging* 12:926. 10.18632/aging.102926 32191631 PMC7138589

[B23] Huang D. W. Sherman B. T. Lempicki R. A. (2009). Systematic and integrative analysis of large gene lists using DAVID bioinformatics resources. *Nat. Protoc.* 4 44–57. 10.1038/nprot.2008.211 19131956

[B24] Huang S. Zhang B. Fan W. Zhao Q. Yang L. Xin W. (2019). Identification of prognostic genes in the acute myeloid leukemia microenvironment. *Aging* 11 10557–10580. 10.18632/aging.102477 31740623 PMC6914404

[B25] Jiang Y. Wang C. Zhou S. (2020). Targeting tumor microenvironment in ovarian cancer: premise and promise. *Biochim. Biophys. Acta Rev. Cancer* 1873:188361. 10.1016/j.bbcan.2020.188361 32234508

[B26] Li Y. Jia J.-H. Kang S. Zhang X.-J. Zhao J. Wang N. (2009). The functional polymorphisms on promoter region of matrix metalloproteinase-12, -13 genes may alter the risk of epithelial ovarian carcinoma in Chinese. *Intern. J. Gynecol. Cancer* 19 129–133. 10.1111/IGC.0b013e31819a1d8e 19258954

[B27] Li Y. Wang H.-C. Wang J.-S. Sun B. Li L.-P. (2020). Chemokine receptor 4 expression is correlated with the occurrence and prognosis of gastric cancer. *FEBS Open Biol.* 10 1149–1161. 10.1002/2211-5463.12864 32306562 PMC7262922

[B28] Ling B. Huang Z. Huang S. Qian L. Li G. Tang Q. (2020). Microenvironment analysis of prognosis and molecular signature of immune-related genes in lung adenocarcinoma. *Oncol. Res.* 10.3727/096504020x15907428281601 32471520 PMC7962936

[B29] Liu C.-F. Liu S.-Y. Min X.-Y. Ji Y.-Y. Wang N. Liu D. (2014). The prognostic value of CXCR4 in ovarian cancer: a meta-analysis. *PLoS One* 9:e92629. 10.1371/journal.pone.0092629 24658065 PMC3962452

[B30] Liu H. Shi J. Cai Z. Huang Y. Lv M. Du H. (2020). Evolution and domestication footprints uncovered from the genomes of Coix. *Mol. Plant* 13 295–308. 10.1016/j.molp.2019.11.009 31778842

[B31] Luan F. Chen W. Chen M. Yan J. Chen H. Yu H. (2019). An autophagy-related long non-coding RNA signature for glioma. *FEBS Open Biol.* 9 653–667. 10.1002/2211-5463.12601 30984540 PMC6443865

[B32] Mahal B. A. Alshalalfa M. Zhao S. G. Beltran H. Chen W. S. Chipidza F. (2020). Genomic and clinical characterization of stromal infiltration markers in prostate cancer. *Cancer* 126 1407–1412. 10.1002/cncr.32688 31905251 PMC7332205

[B33] Matsuo K. Sheridan T. B. Yoshino K. Miyake T. Hew K. E. Im D. D. (2012). Significance of lymphovascular space invasion in epithelial ovarian cancer. *Cancer Med.* 1 156–164. 10.1002/cam4.31 23342265 PMC3544453

[B34] Metsalu T. Vilo J. (2015). ClustVis: a web tool for visualizing clustering of multivariate data using principal component analysis and heatmap. *Nucleic Acids Res.* 43 W566–W570. 10.1093/nar/gkv468 25969447 PMC4489295

[B35] Mizuno H. Kitada K. Nakai K. Sarai A. (2009). PrognoScan: a new database for meta-analysis of the prognostic value of genes. *BMC Med. Genom.* 2:18. 10.1186/1755-8794-2-18 19393097 PMC2689870

[B36] Muqaku B. Pils D. Mader J. C. Aust S. Mangold A. Muqaku L. (2020). Neutrophil extracellular trap formation correlates with favorable overall survival in high grade ovarian cancer. *Cancers* 12:505. 10.3390/cancers12020505 32098278 PMC7072166

[B37] Pan H. Lu L. Cui J. Yang Y. Wang Z. Fan X. (2020). Immunological analyses reveal an immune subtype of uveal melanoma with a poor prognosis. *Aging* 12 1446–1464. 10.18632/aging.102693 31954372 PMC7053626

[B38] Piperi C. Papavassiliou K. A. Papavassiliou A. G. (2019). Pivotal role of STAT3 in shaping glioblastoma immune microenvironment. *Cells* 8:1398. 10.3390/cells8111398 31698775 PMC6912524

[B39] Pu N. Chen Q. Gao S. Liu G. Zhu Y. Yin L. (2019). Genetic landscape of prognostic value in pancreatic ductal adenocarcinoma microenvironment. *Ann. Transl. Med.* 7:645. 10.21037/atm.2019.10.91 31930046 PMC6944582

[B40] Qian J.-X. Yu M. Sun Z. Jiang A.-M. Long B. (2020). A 17-gene expression-based prognostic signature associated with the prognosis of patients with breast cancer: a STROBE-compliant study. *Medicine* 99:e19255. 10.1097/md.0000000000019255 32282693 PMC7220332

[B41] Qu Y. Cheng B. Shao N. Jia Y. Song Q. Tan B. (2020). Prognostic value of immune-related genes in the tumor microenvironment of lung adenocarcinoma and lung squamous cell carcinoma. *Aging* 12 4757–4777. 10.18632/aging.102871 32209727 PMC7138544

[B42] Ramonell K. M. Zhang W. Hadley A. Chen C. Fay K. T. Lyons J. D. (2017). CXCR4 blockade decreases CD4+ T cell exhaustion and improves survival in a murine model of polymicrobial sepsis. *PLoS One* 12:e0188882. 10.1371/journal.pone.0188882 29232699 PMC5726761

[B43] Shimizu A. Sawada K. Kimura T. (2020). Pathophysiological role and potential therapeutic exploitation of exosomes in ovarian cancer. *Cells* 9:814. 10.3390/cells9040814 32230983 PMC7226729

[B44] Thuwajit C. Ferraresi A. Titone R. Thuwajit P. Isidoro C. (2018). The metabolic cross-talk between epithelial cancer cells and stromal fibroblasts in ovarian cancer progression: autophagy plays a role. *Med. Res. Rev.* 38 1235–1254. 10.1002/med.21473 28926101 PMC6032948

[B45] Truxova I. Kasikova L. Hensler M. Skapa P. Laco J. Pecen L. (2018). Mature dendritic cells correlate with favorable immune infiltrate and improved prognosis in ovarian carcinoma patients. *J. Immunother. Cancer* 6:139 10.1186/s40425-018-0446-443 PMC6288908 30526667

[B46] Wang X. Wang H. Wei X. Wang A. Wen L. Wang L. (2018). Effect of CXCR4 silencing with shRNA on MAPK signaling in ovarian cancer. *Oncol. Lett.* 15 10026–10030. 10.3892/ol.2018.8550 29805693 PMC5958742

[B47] Wu M. Shang X. Sun Y. Wu J. Liu G. (2020). Integrated analysis of lymphocyte infiltration-associated lncRNA for ovarian cancer via TCGA, GTEx and GEO datasets. *PeerJ* 8:e8961. 10.7717/peerj.8961 32419983 PMC7211406

[B48] Xie P. Ma Y. Yu S. An R. He J. Zhang H. (2019). Development of an immune-related prognostic signature in breast cancer. *Front. Genet.* 10:1390. 10.3389/fgene.2019.01390 32047513 PMC6997532

[B49] Xie X.-L. Zhu H.-X. Li Y.-M. Chen D.-T. Fan T.-Y. (2020). Differential expression of AURKA/PLK4 in quiescence and senescence of osteosarcoma U2OS cells. *Cell Cycle* 19 884–894. 10.1080/15384101.2020.1731054 32200684 PMC7217361

[B50] Yan H. Qu J. Cao W. Liu Y. Zheng G. Zhang E. (2019). Identification of prognostic genes in the acute myeloid leukemia immune microenvironment based on TCGA data analysis. *Cancer Immunol. Immunother.* 68 1971–1978. 10.1007/s00262-019-02408-2407 31650199 PMC11028253

[B51] Yang H. Zhao K. Kang H. Wang M. Wu A. (2020). Exploring immune-related genes with prognostic value in microenvironment of breast cancer from TCGA database. *Medicine* 99:e19561. 10.1097/md.0000000000019561 32243373 PMC7220520

[B52] Yang S. Liu T. Hongmei O. Wang Y. Chen H. Zhang X. (2020). Comprehensive analysis of prognostic immune-related genes in the tumor microenvironment of cutaneous melanoma. *J. Cell. Physiol.* 235 1025–1035. 10.1002/jcp.29018 31240705

[B53] Yin M. Li X. Tan S. Zhou H. J. Ji W. Bellone S. (2016). Tumor-associated macrophages drive spheroid formation during early transcoelomic metastasis of ovarian cancer. *J. Clin. Invest.* 126 4157–4173. 10.1172/jci87252 27721235 PMC5096908

[B54] Yin X. Wu L. Yang H. Yang H. (2019). Prognostic significance of neutrophil-lymphocyte ratio (NLR) in patients with ovarian cancer: a systematic review and meta-analysis. *Medicine* 98:e17475. 10.1097/md.0000000000017475 31702609 PMC6855616

[B55] Zhang C. Li Z. Qi F. Hu X. Luo J. (2019). Exploration of the relationships between tumor mutation burden with immune infiltrates in clear cell renal cell carcinoma. *Ann. Transl. Med.* 7:648. 10.21037/atm.2019.10.84 31930049 PMC6944593

[B56] Zhang F.-P. Huang Y.-P. Luo W.-X. Deng W.-Y. Liu C.-Q. Xu L.-B. (2020). Construction of a risk score prognosis model based on hepatocellular carcinoma microenvironment. *World J. Gastroenterol.* 26 134–153. 10.3748/wjg.v26.i2.134 31969776 PMC6962430

[B57] Zhang R. Wu J. Yang Y. Xia D. Li J. Quan H. (2020). Donor polymorphisms of Rap1A rs494453 contribute to a higher risk of hepatocellular carcinoma recurrence following liver transplantation. *J. Cancer* 11 3082–3088. 10.7150/jca.39712 32226523 PMC7086244

[B58] Zhang Z. Li Z. Liu Z. Zhang X. Yu N. Xu Z. (2020). Identification of microenvironment-related genes with prognostic value in clear cell renal cell carcinoma. *J. Cell. Biochem.* 121 3606–3615. 10.1002/jcb.29654 31961022

[B59] Zhao X. Xu M. Cai Z. Yuan W. Cui W. Li M. D. (2019). LIFR, PIK3R1Identification of, and as novel prognostic signatures in gallbladder cancer using network-based module analysis. *Front. Oncol.* 9:325. 10.3389/fonc.2019.00325 31119098 PMC6504688

[B60] Zheng H. Zhang G. Zhang L. Wang Q. Li H. Han Y. (2020). Comprehensive review of web servers and bioinformatics tools for cancer prognosis analysis. *Front. Oncol.* 10:68. 10.3389/fonc.2020.00068 32117725 PMC7013087

[B61] Zhu X.-M. Sun W.-F. (2017). Association between matrix metalloproteinases polymorphisms and ovarian cancer risk: a meta-analysis and systematic review. *PLoS One* 12:e0185456. 10.1371/journal.pone.0185456 28957437 PMC5619784

